# Supplementary material for: Are carbon emissions trading and green financial instruments synergistic? -Comprehensive quantitative research based on content analysis
Source: PLoS One. 2024 Mar 7;19(3):e0298601. doi: 10.1371/journal.pone.0298601 (PMC10919601; doi:10.1371/journal.pone.0298601)
Supplement: S1 Data — (ZIP) [file pone.0298601.s001.zip › Data/地级市Z_F工作报告.zip!/─■╧─/╓╨╬└╩╨/└┤╫╘╣1⁄2╓┌║┼í╛┬φ┐╦╩2╛▌═°í┐_2014.docx]

各位代表：

现在，我代表市人民政府，向大会作工作报告，请予审议，并请政协委员和列席人员提出意见。

2013年工作回顾

2013年是新一届政府开局之年，也是全市经济社会发展取得显著成效的一年。在自治区党委、政府和市委的坚强领导下，在市人大、政协的监督支持下，在广大干部群众的团结拼搏下，市政府坚持以科学发展观为指导，深入实施“旅游优先发展、外煤进宁、城市化带动、扶贫攻坚”四大战略，全力以赴稳增长、调结构、惠民生、促和谐，全市经济社会保持了良好发展态势，圆满完成了市三届人大一次会议确定的各项目标任务。预计实现地区生产总值278亿元，增长11%；地方公共财政预算收入17.1亿元，增长15.8%；固定资产投资310亿元，增长26%；社会消费品零售总额50.4亿元，增长14.5%；城镇居民人均可支配收入20082元，增长12.4%；农民人均纯收入6674元，增长12.6%。一年来，主要做了八个方面工作：

一、坚持创新发展，旅游产业快速提升

创新机制驱动。注重顶层设计，委托专业机构，聘请知名专家，编制了中卫市旅游产业发展总体规划、沙坡头旅游经济开发试验区规划和中卫市旅游新镇规划。出台财政、税收、金融等22条优惠政策，获批发行旅游企业债券7亿元，中卫中青旅有限公司挂牌运营，与港中旅初步达成合资合作框架协议。实施旅游标准化体系建设，全国第二批旅游标准化试点城市顺利通过自治区验收，沙坡头被评为“国家生态旅游示范区”。

重点项目拉动。实施沙坡头景区提质扩容、金沙岛改造提升、南长滩千年党项民俗村改造、腾格里湿地生态修复等28个重点项目，新建南长滩拓跋寨、中宁枸杞文化博览中心、海原民族文化产业园等16个旅游项目。沙坡头景区新增直升机、游艇等高端旅游项目，完善旅游接待服务和交通组织系统，旅游业态日渐丰富，产业链条不断延伸。全年接待游客285万人（次），增长21.7%，实现旅游总收入22.4亿元，增长22%。

宣传营销推动。对接客源市场开通旅游包机，102架包机飞抵中卫，51列旅游专列抵达沙坡头。与30家旅行商、32家电视媒体、102家网站合作宣传推介中卫，中卫荣登“2013旅游业最美中国榜”，被新华网评为“最美中国·民俗民族风情旅游目的地城市”。

二、坚持跨越发展，新型工业持续增长

全力保工业增长。建立工业运行监测月报制度，实行市级领导包抓“三个十”责任制。投入5200万元，实施电价补贴、担保贴息等扶持政策，帮企业解困，促企业发展。规模以上工业企业达100家，产值过亿元企业51家，规上工业增加值持续高位增长，预计实现工业增加值106亿元，增长17%。

加快建设重点项目。完成甘塘能源储备转运基地规划，甘武铁路二线获国家发改委批复，通道建设取得突破。新建、续建工业项目52个，完成投资159亿元，锦宁巨科铝板深加工、宁钢二期技改等18个项目建成投产，新增产值28亿元；中宁景津集团60万吨金属锰一期、重庆紫光10万吨蛋氨酸等32个项目开工建设，投产后可新增产值216亿元；以光伏和风能为主的新能源产业发展迅速，保利协鑫等39家新能源企业入驻中卫，在建和建成的新能源项目规模达1790兆瓦。

大力实施招商引资。以“前店后厂”创新模式成功实现与美国亚马逊签约，宁夏中关村科技产业园挂牌成立。引入宽带资本组建西部云基地科技有限公司，完成数据中心选址。成功引进3000万吨煤分质利用、中宁光热电一体化、海原中银绒业等重点项目，大力推进中电投2×350兆瓦热电联产机组等招商项目79个，到位资金266亿元，增长21%。

切实抓好节能减排。制订节能环保对标工作方案，完成天元锰业20万吨电解金属锰热动力节能技改等项目6个，实施宁钢集团脱硫工程治理、城市污水处理、工业废水治理、农业面源减排等项目33个，淘汰落后产能1万吨，万元GDP综合能耗下降2.4%，四项主要污染物削减完成自治区下达年度指标。

三、坚持统筹发展，城乡一体化加快推进

城市更加宜居宜业。大力实施街巷改造、深度保洁、绿化亮化、秩序建设、精品开发“五大战役”和城市“六创”活动，国家园林城市顺利通过验收。开工建设“大河之舞”主题文化公园、沙坡头水镇等精品工程，拆除市区14个片区所有集中连片棚户区，新建、改造鼓楼东西街和南苑路等城市道路37条54公里，改造城市广场5个，填平补齐一大批城市公共设施。城市管理实现精细化、常态化和制度化，“以克论净”深度保洁经验得到自治区党委、政府肯定并在全区推广。

新农村建设扎实推进。实施农村危房改造、垃圾收处、村容村貌改善、农村消防体系、天然气进农户“五大”工程和主干道路大整治大绿化工程，修建农村公路564.5公里，建成农民新居6处，整治旧庄点74个，改造农村危房11699户，天然气入户2000户，组建乡镇消防队15个。沙坡头区农村垃圾全部实现户分类、村收集、镇转运、市集中处理，农村人居环境明显改善。

带动三产不断繁荣。编制完成中卫市现代物流业发展规划，开工建设中卫、中宁黄河公路大桥改扩建、孟家湾至北长滩公路、黑城至海原高速公路等路网工程。四季鲜农产品批发市场投入运营，全民创业城、城市商贸综合体主体完工，中宁陆路口岸封关运营。石嘴山银行中卫分行挂牌设立，全市金融机构贷款余额达309.8亿元，增长24.1%。金融、房地产等现代服务业健康发展，预计实现第三产业增加值109亿元，增长8%。

四、坚持集约发展，现代农业形成规模

特色农业提质增效。建成硒砂瓜品质品牌核心保护区20万亩，创建全国绿色枸杞标准化生产基地10.4万亩，发展高垄管道灌溉马铃薯3万亩、供港蔬菜3万亩。培育农产品加工龙头企业17家，新注册农民专业合作社508个。中宁县被评为“国家级蔬菜栽培标准化示范区”，宁夏红“蛇龙珠”葡萄酒荣获“中国年度十佳葡萄酒”。

草畜产业扩规提质。出台奶（肉）牛产业发展扶持政策和促进鸡产业健康发展意见，种植优质饲草120万亩，改扩建奶（肉）牛、生猪养殖园区62个，肉牛、羊只、生猪饲养量分别达25万头、196万只、106万头，奶牛存栏4万头。

农业基础不断夯实。实施沙坡头南北干渠及灌区节水改造等农田水利项目82个，建设高标准农田11.9万亩，改造中低产田5.9万亩，改善灌溉面积57万亩，新增旱作基本农田4.3万亩。实施土地整理项目7个，整理土地17.5万亩。出台中卫市政策性农业保险若干规定，农业保险实现全覆盖，粮食生产实现10连丰。

五、坚持全面发展，社会事业不断繁荣

加快发展教育事业。促进义务教育均衡发展，改造农村校舍80所20.5万平方米，建成中卫九小、中宁十小、海原四中等中小学校5所。落实“三免一补”资金1.13亿元，惠及学生26.7万人。加快发展幼儿教育，新建、改扩建幼儿园24所，学前一年幼儿毛入园率达94.3%（沙坡头区达到96.4%）。大力发展职业教育，中卫职业技术学校与区内5所高职院校开展合作办学，暨南大学5年内定向为中卫培养硕士研究生30名。编制中卫市人力资源开发利用十年规划，建成市级企业研发机构7家，新建院士工作站2个，实施联合国宁夏沙漠化防治等科技项目36个，申请专利107件。

繁荣文化体育事业。着力加强文化建设，深入开展文化惠民工程，建设数字“农家书屋”452个，放映农村数字电影2616场，文化下乡演出830场（次）。建成村级体育健身广场20个，开展大型全民健身活动13次。推动文化旅游融合发展，成功举办第12届环青海湖国际公路自行车赛中卫赛段活动，群众精神文化生活日益丰富。

深化医药卫生体制改革。实行医药分家管理模式试点，推行社区和基层医疗机构药品零差率销售。建成市人民医院医疗中心、4个乡镇卫生院门诊楼、177所标准化村卫生室，群众医疗需求基本满足。加强人口计生工作，稳定低生育水平，人口出生率控制在自治区下达指标之内，沙坡头区被评为“全国人口计生阳光统计示范单位”。

六、坚持和谐发展，民计民生显著改善

健全社会保障体系。新增财力70%用于改善民生，年初确定的10件民生实事全部完成。城乡居民社会养老、医疗保险统筹覆盖面不断扩大，基础养老金标准和住院医疗报销比例进一步提高，社会保障卡持卡率达95%以上。健全社会救助体系，城乡低保、五保供养、孤儿养育、高龄津贴、重点优抚对象、重度残疾人保障实现全覆盖。新建创业示范基地10个，提供新就业岗位5660个，转移农村劳动力18.8万人，农民工工资“一卡通”覆盖率达95%。开工建设保障性住房12449套78万平方米，保障房受益面进一步扩大。

加快推进扶贫开发。实施重点村“整村推进”42个，开工建设生态移民项目区8个，建成移民住房1593套，安置移民2337户10282人。移民区种植枸杞1.4万亩、苹果0.8万亩，养殖肉牛5460头，山区2.3万人稳定脱贫，6.8万贫困人口生产生活得到明显改善。

加强社会管理创新。扎实推进“平安中卫”建设，建设智能图控、智能交通工程等立体化治安防控体系，整治“三无”小区，依法严厉打击邪教、传销等违法犯罪活动，群众安全感不断增强。加强市区摩托车管制，摩托车上牌率达95%，规范了城市秩序。建立餐饮服务食品无遗漏监管机制，餐饮卫生明显改善。建成安全生产在线智能视频监控系统，153家生产经营单位850处重点部位实现即时监管，全市安全生产形势平稳。深化信访“五联动”工作机制，实行市级领导包抓重点信访案件制度，建立法律援助全覆盖体系，依法加强宗教事务管理，全市民族和睦、宗教和顺、社会和谐。

七、坚持可持续发展，生态建设成效明显

实施生态修复项目。重点实施腾格里沙漠湿地修复、工业园区绿化、南华山水源涵养林、移民迁出区生态修复等项目，新增造林31.8万亩。完成市区“36纵26横”大绿量、多层次、全覆盖绿化改造工程，城市绿地率和绿化覆盖率分别达37%和39%。中卫市荣获“全国十佳生态文明建设示范城市”和“2013年度中国生态文明价值城市”。

加快发展循环经济。积极探索光伏产业生态化重组路径，加强城市水源地保护、水污染防治和大气污染治理，黄河干流中卫出境断面二类水质达标率100%，沙坡头区环境空气质量优良天数达320天。实行生产、流通、消费过程减量化、再利用、资源化，推进绿色低碳发展，沙坡头区成为国家可持续发展实验区，中宁工业园区跻身国家20个循环化改造示范试点园区。

大力推广节水技术。实施污水治理、中水利用等工程，加快节水型社会建设，实行最严格水资源管理。开工建设20万亩高效节水农业示范基地，建成2.7万亩，8万亩正在进行土地流转。加强农村基层水利服务体系建设和农村饮水安全工程管理，高效节水灌溉技术推广成效明显。

八、坚持依法行政，政府自身建设不断加强

推进民主法制建设。自觉接受人大法律监督和政协民主监督，办结人大代表建议38件、政协委员提案222件，办复率100%。积极支持民主党派、工商联、无党派人士参政议政，密切联系工会、共青团、妇联等人民团体。扎实开展“六五”普法，实施国家公职人员行政法规培训考试五年规划，培训1.6万人，沙坡头区被评为“全国法治县（区）创建先进单位”。

切实加强廉政建设。严格执行中央“八项规定”，停建各类楼堂馆所，“三公”经费支出较上年下降27%。实行网上采购，节约资金1.7亿元。成立中卫市国资委，加强国有资产管理，强化审计监督，深入开展工程建设领域突出问题专项治理。严肃查办违纪违法案件121件，给予党纪政纪处分135人，挽回经济损失700余万元。切实加强政风行风建设，群众评议机关和干部作风连续5年列全区5市第一。

不断提升政府效能。减少、合并政府议事协调机构28个，压缩文件43.3%，减少会议34.3%。精简行政审批事项136项，减少行政收费项目39项。主动公开行政审批、财政预算等信息，广泛接受社会监督。实行工业项目行政审批代办和零收费制，综合服务窗口全程代办，所有行政审批“一个窗口进、一个窗口出”一站限时办结，收费全免。实行市长督办副市长和“五定”工作法，市政府常务会议督办重点工作146项。市长信箱解决群众诉求2458件。

全民国防教育、双拥共建、民兵预备役、防震减灾、人民防空、外事侨务、市志档案、邮政通信、气象服务等各项事业取得了新的业绩。

各位代表，过去的一年，全市稳增长收到了明显成效，促发展取得了长足进步，惠民生实现了较大突破，全面建设小康社会迈出了坚实步伐。这是自治区党委、政府和市委总揽全局、正确领导的结果，是市人大、政协支持配合、有效监督的结果，是全市广大干部群众真抓实干、开拓创新的结果，是社会各界凝心聚力、和衷共济的结果。在此，我代表市人民政府，向全市人民，向人大代表、政协委员，向各民主党派、工商联、无党派人士、人民团体，向驻卫部队、武警官兵，向所有关心、支持和参与中卫发展的各界人士、企业家们，表示崇高的敬意和衷心的感谢！

在充分肯定成绩的同时，我们也清醒地认识到，中卫经济社会发展还存在不少矛盾和问题，主要表现在：经济总量较小，发展基础相对薄弱；产业结构不尽合理，节能减排形势严峻；山川发展不平衡，扶贫攻坚任务艰巨；城镇化进程较为缓慢，现代服务业发展滞后；社会治理还需进一步加强，全面建成小康社会任务繁重等。对此，我们要高度重视，采取强力措施，切实加以解决。

2014年主要工作

今年是中卫建市十周年，也是在新的起点上全面深化改革、谋求转型跨越的重要一年。政府工作的总体要求是：坚持以科学发展观为统领，认真贯彻落实党的十八大和十八届三中全会、自治区党委十一届三次全会、自治区“两会”及市委三届四次全体会议精神，以建市十周年为新起点，深入实施“四大战略”，紧紧围绕“1050”（10项行动计划、50个重点项目）工作任务，进一步抓改革、调结构、转方式、惠民生、促和谐，奋力开启中卫二次创业新征程，加快建设开放中卫、富裕中卫、和谐中卫、美丽中卫，为2020年全面建成小康社会奠定坚实基础。

主要预期目标是：地区生产总值增长10.5%左右，规模以上工业增加值增长13%，地方公共财政预算收入增长13%，固定资产投资增长20%，社会消费品零售总额增长13%，城镇居民人均可支配收入增长11%，农民人均纯收入增长12%，人口自然增长率控制在10.05‰以内，城镇登记失业率控制在4.5%以内，单位生产总值能耗下降6%以上，努力完成自治区下达的节能减排任务。

一、深化改革开放，建设开放中卫

进一步解放思想。建设开放中卫，关键是解放思想，核心是树立市场在资源配置中起决定性作用的理念，正确处理好政府和市场的关系，按市场规律办事，该管的坚决管好，不该管的交由市场和社会组织。重点是发挥企业在市场中的主体作用，加快建立现代企业制度，激发企业活力，坚决清除对非公有制经济各种形式的不合理规定，使非公有制经济真正成为经济增长的主力军。关键是各级领导干部要开阔眼界、开阔思路、开阔胸襟，坚持在解放思想中统一思想，深入开展“五破五立”解放思想大讨论，破除急功近利、竭泽而渔的思想，树立环保为先、绿色发展的理念；破除部门至上、各行其是的思想，树立服从大局、服务发展的理念；破除得过且过、不思进取的思想，树立奋发有为、争先进位的理念；破除固步自封、因循守旧的思想，树立开放包容、敢闯敢试的理念；破除坐而论道、怕担责任的思想，树立真抓实干、敢于担当的理念。以更大的决心冲破思想观念的束缚，突破利益固化的藩篱，能改的先改起来，能干的先干起来，能用的办法先用起来，大胆探索、大胆实践、大胆突破，以思想大解放推动中卫大发展。

加快推进改革创新。建设开放中卫，核心是打造“两优”环境，大力实施改革创新行动计划，全面推进行政体制、农村土地产权制度、教育领域综合改革、文化卫生体制、财税金融、社会治理体制等重点领域和关键环节改革，6月前完成市县（区）政府机构改革，加快事业单位分类改革，深化经济体制改革，统筹社会领域改革，创新行政管理方式，增强政府治理能力，把工作重点转到创造良好发展环境、提供优质公共服务、维护社会公平正义上来。加快实施建设服务高地行动计划，推进行政审批制度改革，实行“两个清单”（行政审批事项负面清单、政府权力清单），采取“一个模式”（审批服务“一门受理、多证联办”），坚持“三个一律”（凡负面清单以外、市场机制能够有效调节的经济活动一律取消审批，直接面向基层、面向群众，由基层管理更方便有效的经济社会事项一律下放审批权限，企业核准项目一律实行备案制）。出台中卫市人民政府“两优”行政审批制度和“两优”行政审批轮值主席会议制度，把行政审批事项从229项减到80项，审批时限从最长60天压缩到5天，把多窗口办理合并到一个窗口办理，把诸多的行政审批前置要件改为后置要件，取消所有行政性收费，打造在西部最优、比东部更优的投资发展环境。

不断扩大对外开放。建设开放中卫，突破口是扩大对内对外开放，抢抓建设丝绸之路经济带战略支点的重大机遇，用好中阿合作平台和内陆开放型经济试验区有关政策，加强与阿拉伯国家的合作，加强与北京、新疆、内蒙古等地区的合作，加强与世界500强及大型民营企业的合作。以“前店后厂”模式建设宁夏中关村科技产业园，深化与亚马逊、奇虎360等龙头企业合作，推进西部云基地建设，吸引电子商务、物联网、网络金融、云服务等企业进驻，大力发展信息产业。加快推进甘塘能源储备转运基地建设，争取国家尽快批复规划，启动建设主干道路、变电站、供水工程等基础设施。以“深度合作开发”模式，启动旅游经济开发试验区建设，组建沙坡头旅游经济开发试验区管委会，将旅游经济开发试验区建成集“产、居、商、旅”四位一体的综合性旅游开发区。以“政企合作”模式，创办中阿科技大学，搭建大宗商品O2O交易平台，构筑全方位、多层次对内对外开放新格局。

二、推进经济转型，建设富裕中卫

调整优化产业布局。大力实施产业结构调整行动计划，坚持“环保为先、增量从优、长短结合、淘汰落后”的原则，以“一园一区四基地”为载体，加快实施“3321”（三优先、三推进、二审慎、一淘汰）计划，即优先鼓励发展旅游、物流和信息三个产业，积极稳妥推进疆煤为主的煤炭清洁利用、光伏和风能为主的新能源、特色农产品及其加工三个产业，审慎适度发展冶金、精细化工两个产业，坚决淘汰一批污染严重和能耗居高不下的落后产能，力促经济转型升级。进一步优化投资结构，集中开工建设50个重点项目，主要是中关村科技产业园数据中心等20个工业项目，旅游新镇等10个旅游项目，城市森林公园等8个城市建设项目，高效节水灌溉等5个农业项目，甘武铁路二线等5个交通项目，中阿科技大学等2个社会事业项目，以大项目促进结构调整，推动转型升级。

突出发展新型工业。坚持招大商选优商。健全完善市级领导定点包抓重大招商项目、招商项目准入评估、招商引资联席会议等制度，形成市级领导顶层推动、主管部门牵头负责、县（区）部门协同推进的招商引资新机制。修订完善中卫市招商引资相关政策，积极争取国家和自治区资金、项目及政策支持，严格项目准入机制，变“招商引资”为“招商选资”，年内完成招商引资到位资金300亿元。加快推进重点项目建设。按照新型工业化的要求，积极推进工业结构战略性调整，创设中卫创新研究院，启动运营宁夏中关村科技产业园，开工建设新一代数据中心，建设智慧产业城，抓好云计算配套服务基础设施建设和产业发展。重点推进新疆广汇1000万吨煤分质利用、金沙江40亿立方米煤制天然气、中电投2×350兆瓦热电联产机组、600兆瓦新能源发电等重大项目建设。加快推进重庆紫光10万吨蛋氨酸、中宁景津集团60万吨金属锰二期、锦宁120万吨铝镁合金二期、海原中银绒业羊绒制品等续建项目，努力构建可持续发展的现代工业体系。大力扶持非公有制经济和中小微企业发展。实施“百家成长、千家培育”工程，培育自治区级中小企业100家、销售收入过100亿元企业1家、销售收入过50亿元企业3家。落实鼓励支持中小微企业繁荣发展20条政策，引导民间借贷合法发展，扩大中小微企业贷款规模，力争新增贷款20亿元，提供贷款担保20亿元。市县财政每年安排5000万元用于中小微企业贷款逆向贴息，大力营造非公有制经济和中小微企业快速发展的良好环境。

大力发展现代农业。构建新型农业经营体系。积极推进农村集体土地确权登记颁证，引导农村土地经营权有序流转，集中发展绿色枸杞、硒砂瓜、酿酒葡萄、马铃薯等特色产业。探索试行土地承包经营权担保、抵押、入股，推进家庭农场、龙头企业、专业大户等新型农业经营主体共同发展。积极培育各类农村专业合作组织，扶持宁夏红、早康枸杞、夏华肉食品等农业产业化龙头企业健康发展，不断提高农业生产的规模化、集约化、市场化水平。促进特色农业提质增效。推进20万亩绿色枸杞基地建设，发展枸杞标准化示范园2万亩，加强与特步集团等知名企业合作，推动枸杞产业向品牌化、规模化、深加工方向发展。建设硒砂瓜品质品牌保护区40万亩，建成四季鲜硒砂瓜销售中心。发展质数水稻1万亩、制种玉米4.3万亩，启动10万亩葡萄基地基础设施建设，年内种植酿酒葡萄1万亩。建设设施农业提质增效示范园8个，全市设施农业面积达到20万亩。加快发展草畜产业，新建万头奶牛场2个，千头奶牛场3个、肉牛场2个，全市奶牛存栏4.5万头、肉牛饲养量达到27万头。不断夯实农业发展基础。实施灌区8万亩高效节水农业示范项目，抓好沙坡头南北干渠节水改造、中低产田改造等基础设施建设项目。积极做好香山扬水、甘宁两省中北部扶贫暨生态移民供水、宁夏中南部供水联通等工程前期工作。积极开展沙坡头区“全国气象为民服务‘百县千乡’示范县区”建设，提高气象等自然灾害防御能力。

加快发展现代服务业。优先发展旅游产业。启动旅游经济开发试验区建设，整合旅游资源，完善产业体系，统筹旅游吸引物、接待服务和交通组织三大系统建设。发行7亿元旅游企业债券。开工建设旅游新镇、游客服务中心、大漠风情园等项目。引进同仁堂等一批知名企业，发展旅游养生保健产业。全面推进旅游标准化建设，积极开展城旅一体化改造。启动智慧旅游工程建设，提升旅游市场宣传营销和监管服务水平。积极推进与港中旅等旅游企业的深度合作，推动旅游产业集团化、现代化发展。全年游客人数和旅游总收入同比均增长20%。加快构建现代物流体系。开工建设甘武铁路二线、中卫工业园区铁路专用线及镇罗堡火车站开站项目，积极做好包兰铁路扩能改造、西线高速公路中卫段等项目前期工作，加快实施中卫、中宁黄河大桥和黑城至海原高速公路、迎闫公路、广申大道立交桥等25个重点交通项目。引入专业物流集团落户中卫，加快推进甘塘煤炭物流中心、大西北物流城、中宁陆路口岸、海原工业物流园区、旅游快递系统等建设，完善现代物流体系。大力发展现代服务业。建立大宗商品现货交易所和中心交割库。推动网络升级为骨干节点并实现互联互通，创新“前店后厂”模式，发展云计算等信息服务业。支持具备条件的民间资本依法设立中小型银行，争取创办宁夏中关村银行、中宁民营银行。积极推进金融保险、中介咨询、健康养生、商贸餐饮等产业繁荣发展，服务业增加值同比增长10%。

三、保障改善民生，建设和谐中卫

全面发展社会事业。实施育才引才行动计划，办好人民满意的教育，推进义务教育均衡发展，确保沙坡头区、中宁县义务教育顺利通过自治区验收。全面普及高中阶段教育，促进兴海中学与六盘山高级中学联合办学，提升职业教育层次和水平。开工建设中阿科技大学暨教育园区，年内建成并招生。积极推进人才资源开发利用，建立中卫在外人才信息库，完善人才引进、培养、使用、激励等服务机制。加强公共文化服务，完成博物馆、黄河宫布展，开展“文化下乡”惠民演出800场，举办全国第二届大漠健身运动会等大型文体活动。发展文化产业，推动文化旅游融合发展，着力打造枸杞制品、剪纸刺绣、黄河奇石、沙工艺品等系列特色文化旅游产品。借力西部云基地建设，发展创意设计、数字技术、网络、动漫等文化产业。继续深化医药卫生体制改革，健全村卫生室医疗服务保障体系。扎实做好人口和计划生育工作，落实少生快富项目户1000户，适时启动“单独二孩”政策，促进人口素质提高和长期均衡发展。

加快扶贫开发进程。实施全面小康社会推进行动计划，继续抓好扶贫攻坚和生态移民工作，年内完成海原县“十二五”生态移民搬迁任务的80%。大力培育生态移民区后续产业，深入实施“5351”生态移民就业计划，加强移民区社会治理和公共服务，努力实现移民安居乐业。落实扶贫政策，创新扶贫模式，完善定点帮扶，实施重点村“整村推进”96个、村级互助发展资金项目193个，年内减少贫困人口3.3万人。

完善社会保障体系。实施民生实事行动计划，办好15件民生实事。推进创业型城市建设，建立政府激励创业、社会支持创业、劳动者勇于创业新机制，以创业带动就业，城镇新增就业8200人，农村劳动力转移就业15万人。力争基本养老、医疗保险覆盖城乡所有居民，扩大失业、工伤、生育等保险覆盖面，农民工工资“一卡通”覆盖率达95%以上。健全留守儿童、妇女、老人关爱服务和残疾人权益保障等社会救助体系，完善社会养老服务体系，新建、改建敬老院3所。扩大住房保障覆盖面，开工建设保障性住房1.07万套。建立公开规范的住房公积金制度，促进解决城镇职工住房问题。加强物价监管，居民消费价格总水平涨幅控制在4%以内。

创新社会治理机制。深化“平安中卫”建设，构建立体化社会治安防控体系，依法严密防范和惩治各类违法犯罪活动。扎实开展“物业管理年”活动，分步整治“三无”小区。深化“三调联动”大调解和信访“五联动”工作机制，建立涉法涉诉信访依法终结制度，引导群众依法表达合理诉求、维护合法权益。推进军民融合式发展，共创双拥模范城市。依法管理宗教事务，巩固发展新型民族关系。完善重大决策社会稳定风险评估机制，健全突发公共事件应急管理体系，提高突发事件预防、预警和应急处置能力。发挥安全生产在线视频监控平台作用，抓好安全生产和食品安全工作，坚决防止重特大事故发生，确保人民群众生命财产安全。

四、加强生态保护，建设美丽中卫

统筹城乡一体化发展。深入实施“城市化带动”战略，抓好“四城八镇十村”建设，创新城市建设管理机制，围绕绿化、净化、亮化、美化，提升市区、中宁县城、海原县城和海兴开发区的规划、建设、管理水平。实施中卫第二水厂、中宁工业固废填埋场、海兴开发区污水处理厂等项目，改造二干渠，建设城市第二景观水系，建成城市文化广场和沙坡头水镇。完成市区1456户棚户区改造，新建、改扩建城市道路31条66.5公里，敷设供水管网61.2公里，新增排水管网44.2公里，铺设供热管网41.8公里。继续推进城市“六创”，深化“以绿为美”美丽绿化机制和“以克论净”深度保洁机制，合理布局城市建筑群，控制高层建筑，提高城市空气质量，改善人居环境。制定出台规范与限制城市摩托车的11条规定、静态交通管理的12条规定，规范市区交通秩序，建设机动车智能停车位，实行智能刷卡停车，实现有序停车，规范商户有序经营，提升城市形象。重点改造建设迎水桥镇、石空镇、七营镇等8个特色小城镇，提升城镇产业承载和人口吸纳能力。抓好沙坡头区迎水桥镇迎水村、中宁县恩和镇朱台村、海原县郑旗乡郑旗村等10个“幸福村庄”改造整治工程，完成农房改造8100户，积极推动基础设施、公共服务逐步向农村延伸拓展，提升新农村建设水平。加快推进户籍制度改革，统筹城镇就业、社会保障等工作，加快具备条件的农民变市民步伐。

加强生态环境保护和治理。实施美丽中卫建设行动计划，编制中卫市2014—2023年绿化规划，科学划定生态修复主体功能区。加强生态文明制度建设，建立更加严格的环境准入制度，制定差异化产业扶持政策，对不符合国家环保政策的项目一律不支持、不批准。加强水源地保护，开工建设城市森林公园，完成一期造林8000亩。实施“大河之舞”主题文化公园西延伸段工程、沙漠光伏生态产业园、南华山水源涵养林、腾格里沙漠湿地修复、移民迁出区生态修复等重点项目，巩固扩大禁牧封育、退耕还林还草成果。加强大气污染防治和固体废弃物定点集中无害化处理，建立PM2.5监测体系和定期发布环境质量公告制度，打造生态中卫。

扎实推进节能减排。实施工业园区节能环保行动计划，加快推进企业节能技术改造，大力推动中卫、中宁工业园区发展循环经济。建立更加严格的节能减排制度，推行合同能源管理，严格控制新上“两高一资”项目，对新上项目能评不达标的一律禁止。出台中卫市节能减排对标管理具体规定，建立节能对标管理体系和环境质量全面量化控制体系，加快淘汰落后产能，实施重点耗能、排污企业许可证制度和终生责任追究制，对能耗和污染严重超标的企业一律关停。

集约节约利用资源。实施节水型社会建设行动计划，推行最严格的水资源管理制度，探索水权转换支持工业发展模式，主攻农业节水，控制工业耗水，规范生活用水，提倡全民节水，提高水资源利用效率和效益。推进土地资源节约集约利用，严守耕地保护红线，确保全市耕地保有量363万亩，基本农田巩固在292万亩。加强矿产资源管理，促进矿产资源有序开发、合理利用。推进污水、生活垃圾、危险废物处理等环保设施建设，强化农村面源污染治理，推广使用农村沼气等清洁能源。加快新能源和节能产品推广运用，推行清洁生产，实现可持续发展。

五、转变政府职能，建设人民满意政府

建设法治政府。深入推进依法治市，坚持依法行政，建立法律顾问制度。主动接受人大及其常委会的法律监督、政协民主监督，认真办理人大代表建议和政协委员提案。密切联系各民主党派和群众团体，自觉接受社会和舆论监督。广泛开展“六五”普法，深入实施法律“六进”活动，切实加强行政执法监管，开展行政复议委员会试点，建立完善困难群体法律援助和司法救助机制，健全社区矫正制度，加快推进法治中卫建设。

建设廉洁政府。落实市委党建提升行动计划，认真履行党风廉政建设责任制，全面推进惩治和预防腐败体系建设，充分发挥监察、审计、财政的监督职能，严查各类违纪违法案件。切实加强对工程建设、公共资源交易、政府采购等重点领域、重大项目、重要资金监管，始终保持查办案件的高压态势，把权力关进制度的“笼子”里。深入开展纠风治乱和专项治理，建立完善涉农资金动态监管机制，坚决纠正损害群众利益行为。认真贯彻落实中央“八项规定”和《党政机关厉行节约反对浪费条例》《党政机关国内公务接待管理规定》等，一律停建楼堂馆所，坚决清理违规办公用房和公务用车，从严控制出国出境，规范考察、出差等活动，大幅压缩“三公”经费，降低行政运行成本。切实加强政风行风建设，广泛开展群众评议机关和干部作风活动，以反腐倡廉的实际成效取信于民。

建设高效政府。健全重大事项集体决策、专家咨询、社会公示、听证评估等制度，使政府决策科学、透明、高效。不断提升政府决策水平和执行力，大力发展电子政务，压缩行政审批事项，简化行政审批程序，构建高效便捷的政务服务体系。以解决“四风”为突破口，扎实开展群众路线教育实践活动，坚决遏制迎来送往、文山会海、铺张浪费，大幅精简文件、压缩会议，文件和会议数量再压缩30%。继续推行市长督办副市长、政府常务会议督办重点工作和“五定”工作法，用干部的辛苦指数换取群众的幸福指数。进一步严格绩效管理，完善考核评价体系，形成任务定责、行政督责、失职追责的问责体系，用实干推动中卫发展，用实干造福全市人民。

各位代表，建设开放中卫、富裕中卫、和谐中卫、美丽中卫，是时代的召唤，更是全市人民的热切期盼！让我们在自治区党委、政府和市委的坚强领导下，在全面建成小康社会宏伟目标的激励下，抢抓新机遇，勇挑新重担，创造新业绩，完成新使命，努力开创中卫更加美好的明天！
